# Antimutagenicity of Methanolic Extracts from *Anemopsis californica* in Relation to Their Antioxidant Activity

**DOI:** 10.1155/2014/273878

**Published:** 2014-07-23

**Authors:** Carmen Lizette Del-Toro-Sánchez, Nereyda Bautista-Bautista, José Luis Blasco-Cabal, Marisela Gonzalez-Ávila, Melesio Gutiérrez-Lomelí, Myriam Arriaga-Alba

**Affiliations:** ^1^Centro Universitario de la Ciénega, Universidad de Guadalajara, Avenida Universidad 1115, 47820 Ocotlán, JAL, Mexico; ^2^Microbiology Research Laboratory, Research Department, Hospital Juárez de México, Avenida Instituto Politécnico Nacional No. 5160, 07760 Ciudad de México, DF, Mexico; ^3^Centro de Investigación y Asistencia en Tecnología y Diseño del Estado de Jalisco, A.C. Avenida Normalistas No. 800, Col. Colinas de la Normal, 44270 Guadalajara, JAL, Mexico

## Abstract

*Anemopsis californica* has been used empirically to treat infectious diseases. However, there are no antimutagenic evaluation reports on this plant. The present study evaluated the antioxidant activity in relation to the mutagenic and antimutagenic activity properties of leaf (LME) and stem (SME) methanolic extracts of* A. californica* collected in the central Mexican state of Querétaro. Antioxidant properties and total phenols of extracts were evaluated using DPPH (1,1-diphenyl-2-picrylhydrazyl) and Folin-Ciocalteu methods, respectively. Mutagenicity was evaluated using the Ames test employing *Salmonella enterica* serovar Typhimurium strains (TA98, TA100, and TA102), with and without an aroclor 1254 (S9 mixture). Antimutagenesis was performed against mutations induced on the Ames test with MNNG, 2AA, or 4NQO. SME presented the highest antioxidant capacity and total phenolic content. None of the extracts exhibited mutagenicity in the Ames test. The extracts produced a significant reduction in 2AA-induced mutations in *S. typhimurium* TA98. In both extracts, mutagenesis induced by 4NQO or methyl-N′-nitro-N-nitrosoguanidine (MNNG) was reduced only if the exposure of strains was <10 *μ*g/Petri dish.* A. californca* antioxidant properties and its capacity to reduce point mutations render it suitable to enhance medical cancer treatments. The significant effect against antimutagenic 2AA suggests that their consumption would provide protection against carcinogenic polycyclic aromatic compounds.

## 1. Introduction

Antimutagenic studies are a good alternative for obtaining important information that explores the possibilities for reducing the genotoxic risk exposure to genotoxic compounds. Many studies of natural plants, such as* Stevia pilosa*,* Stevia eupatoria*, and* Castela texana*, have exhibited antimutagenic and antigenotoxic properties [1, 2]. Among these plants, it has been reported that antioxidants, a mixture of components that can reduce reactive oxygen species (ROS), induce mutagens or improve alkylated DNA damage [3, 4]. Phenolic compounds serve to improve the antioxidant properties of the plants; thus, they have attracted special attention [5].

The mechanism of mutagenesis is complex. However, many mutagens and carcinogens may act through the generation of ROS [6]. ROS may play a major role as endogenous initiators of degenerative processes, such as DNA damage and mutation. Therefore, the discovery and the exploration of compounds possessing antioxidant and mutagenic properties are of great practical and therapeutic significance [7].

The* Salmonella enterica* serovar Typhimurium Ames method has been recommended as the first step for initiating a genotoxic risk evaluation for new products to which humans are exposed, mainly for therapeutic drugs. This method has been accepted by several international organizations and these organizations occasionally suspend research on new products simply because the product in question produced a positive result on the Ames test [8–12].

*Anemopsis californica* is a plant of the Saururaceae family; its common name in Mexico is* hierba mansa* or* hierba del manzo* [13, 14]. It grows in the southwestern states of the US and in the northern and central states of Mexico [15–17]. In the state of Querétaro (a central state in Mexico), the plant has been found in the swamps of Juriquilla at an altitude of 1,900 m [18]. It has been widely used in traditional medicine to treat several medical conditions. In some cases, it has been recommended as a treatment for gastric problems, dysmenorrhea, and venereal diseases [19–22], as an anticarcinogenic [23, 24], and as an antibacterial [25]. The variety of components found in this plant may explain its wide therapeutic uses, some of which are empirically proven [26]. However, to date no antimutagenic evaluations of* A. californica* have been conducted. In this study, we report the evaluation of the antioxidant activity in relation to the mutagenic and antimutagenic properties of LME and SME* A. californica* collected in the state of Querétaro, Mexico. These studies were performed in order to know the mutagenic and antimutagenic properties of this plant.

## 2. Methods

### 2.1. Chemicals, Strains, and Animals

4-Nitroquinoline-1-oxide (4NQO), picrolonic acid (PA), methyl-N′-nitro-N-nitrosoguanidine (MNNG), 2-aminoanthracene (2AA), cyclophosphamide (CP), 1,1-diphenyl-2-picrylhydrazyl (DPPH), and mitomycin C were obtained from Sigma (St. Louis, MO, USA) and aroclor 1254 was purchased from Supelco (Bellefonte, PA, USA).* S. typhimurium* strains TA98 (*hisD3053*,* rfa*,* pKm101, and uvrB*), TA100 (*hisG46, rfa*,* pKm101*, and* uvrB)*, and TA102 (*hisG 8976, rfa, pkM101, hisG428, pQ1, and uv*rB+) were kindly donated by Dr. Bruce Ames, University of California, Berkeley. The S9 homogenate fraction of rat liver induced with aroclor 1254 was prepared as described by Maron and Ames [27].

### 2.2. *Anemopsis californica* Extract Preparation

Leaves and stems of* Anemopsis californica* were collected from swamp soil in Querétaro (Mexico) in February of 2012; they were cut and dried separately for 48 h at 37°C. The dried material was ground into small particles. Five grams of each sample, either leaves or stems, was mixed with 15 mL of methanol and homogenized with an Ultraturrax (T 25 DS1 digital homogenizer) for 1 min. Afterwards, the sample was ultrasonicated (Bransonic, 151-DTH) at 4°C for 15 min. The supernatant was collected after centrifugation at 4,000 rpm for 15 min at 4°C. The extraction procedure was repeated for a second time. The combined supernatants were evaporated under vacuum conditions at 45–50°C (Heidolph Rotavapor, 4003 VAC Senso T) to dryness. The dry extract was solubilized in methanol to a final concentration of 14 mg/mL [25]. The final extracts were denominated leaf and stem methanolic extracts (LME and SME), respectively.

### 2.3. Antioxidant Capacity Test

The antioxidant activities were determined using DPPH as a free radical [28]. Different methanolic dilutions of LME and SME (0.1, 0.2, 0.43, 0.87, 1.75, 3.5, 7.0, and 14 mg/mL) were tested. A volume of 0.1 mL of these extract solutions was added to 3.9 mL of a 6 × 10^−5^ mol/L DPPH solution. The decrease in absorbance was determined at 515 nm at 0 and 30 min under conditions of darkness. The control sample was prepared containing the same volume without any extract. Methanol was used as the blank. All determinations were carried out in triplicate. The results are reported as % of inhibition with the equation

(1) %  of  inhibition   =(initial  absorbance−final  absorbance)initial  absorbance∗100.

### 2.4. Determination of the Total Phenolic Content

Estimation of the total phenolic content was performed as it was reported by others [29–31]; we customized the material for 96-well microplates. LME and SME extracts were diluted in methanol at a ratio of 1 : 200 and 1 : 100, respectively. Gallic acid, prepared in six concentrations ranging from 4 to 20 mg/L, was used as a standard.

Thirty *μ*L of each extract or standard solution, except in a blank probe where only the solvent was used, was added to 150 *μ*L of 0.1 mol/L Folin-Ciocalteu reagent and mixed with 120 *μ*L of sodium carbonate (7.5%) after 10 min. Absorbance at 760 nm was read after 2 h. The phenolic concentration was determined by comparison with the standard calibration curve of gallic acid, and the results are presented as a mean value of triplicate tests. The total phenol value was expressed as mg of gallic acid equivalents (GAE) per g of dry weight (dw).

### 2.5. *In Vitro* Mutagenesis Assays

*S. typhimurium* strains, TA98, TA100, or TA102, were incubated with Oxoid nutrient broth number 2 for 16 h at 37°C/90 rpm. Then, 100 *μ*L of the culture was poured into a sterile tube containing a soft agar with 0.5 mM histidine-biotin as described by Maron and Ames [27]. The tester strains were then treated with 250–1,000 *μ*g/Petri dish of LME or SME, with or without the S9 mixture. As positive controls, TA98 was exposed to 50 *μ*g/Petri dish of (PA) (10 *μ*L of 5 *μ*g/*μ*L of (PA)), without S9, or 10 *μ*g/Petri dish of (2AA) (10 *μ*L of 1.0 *μ*g/*μ*L of (2AA)) with S9, TA100 to 10 *μ*g/Petri dish of MNNG (10 *μ*L of 1.0 *μ*g/*μ*L of MNNG) or 500 *μ*g/Petri dish of CP (10.0 *μ*L of 50.0 *μ*g/*μ*L of CP) with S9, and TA102 with 10 *μ*g/Petri dish of 4NQ0 (10 *μ*L of 1.0 *μ*g/*μ*L of 4NQ0) with or without S9. They were poured into Vogel-Bonner medium and incubated for 48 h at 37°C. Then, histidine revertant colonies were counted with a Fisher-Scientific semiautomatic colony counter. A positive result is given when the number of spontaneous histidine (+) revertants was twice the amount of that of spontaneous reversion.

### 2.6. *In Vitro *Antimutagenesis Assays

Antimutagenesis assays were performed using the Ames test as described by Maron and Ames [27]. The first group of studies was conducted by exposing* S. typhimurium* to the following different mutagens: TA100 to MNNG (5–10 *μ*g/Petri dish),* S. typhimurium* TA98 to 2AA (5–10 *μ*g/dish) with the aroclor 1254-induced S9 mixture (S9 mix), and* S. typhimurium* TA102 to 4NQNO (5–10 *μ*g/Petri dish). Secondly, the three strains were also exposed to 250 or 500 *μ*g of LME and SME without the mutagen and with or without the S9 mix [27]. Finally, antimutagenesis was evaluated, exposing each* S. typhimurium* strain to the appropriate dose of mutagens MNNG, 2AA, or 4NQO, plus LME or SME (250 or 500 *μ*g/Petri dish), under suitable assay conditions as described previously.

### 2.7. Statistical Analysis

The results were evaluated using a two-part analysis with Graphic-Presentation Prism version 2.01 software, comparing the reduction of mutations induced only with the positive control with those exposed to both the positive control and the plant extracts.

## 3. Results

### 3.1. Antioxidant Capacity Test

**Figure 1:**
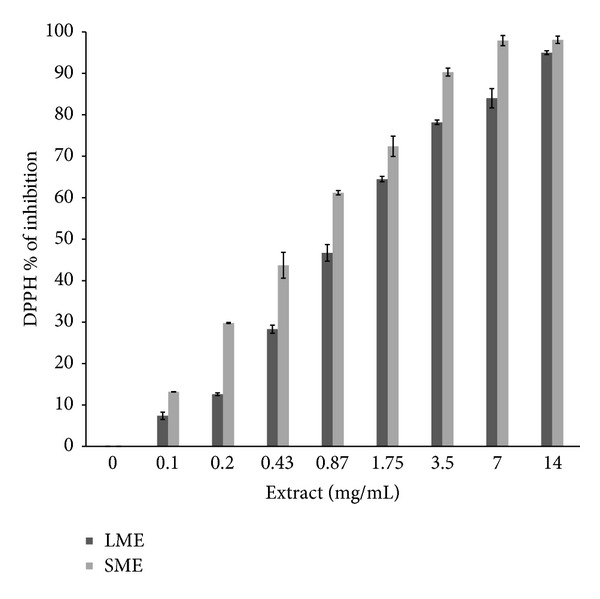
DPPH (1,1-diphenyl-2-picrylhydrazyl), antioxidant evaluation with leaves (LME) and stems (SME) of* A. californica*.

Figure 1 depicts a dose-curve response with respect to an increase of extract concentration. SME presented more antioxidant activity than LME. Highest antioxidant activity was observed in SME at 7 mg/mL while LME was 14 mg/mL (98.1 and 95%, resp.).

### 3.2. Total Phenolic Content

The amount of the total phenolics was found to be very different between the stem and leaf of* A. californica*. SME (79.44 ± 3.50 mg GAE/g of dw) presented higher total phenols than LME (27.84 ± 0.98 mg GAE/g of dw). According to the previously mentioned antioxidant results, there was a correlation between total phenolic content and antioxidant activities.

### 3.3. Mutagenesis

Neither extract (LME and SME) induced basepair mutations on* S. typhimurium* TA100, frameshift mutations on* S. typhimurium* TA98, nor ROS DNA damage in strain TA102. LME induced a twofold increase of spontaneous mutations at the higher doses (1 mg/Petri dish) only with the S9 mixture, but a dose-response curve could not be plotted (Table 1).

### 3.4. Antimutagenesis

**Figure 2:**
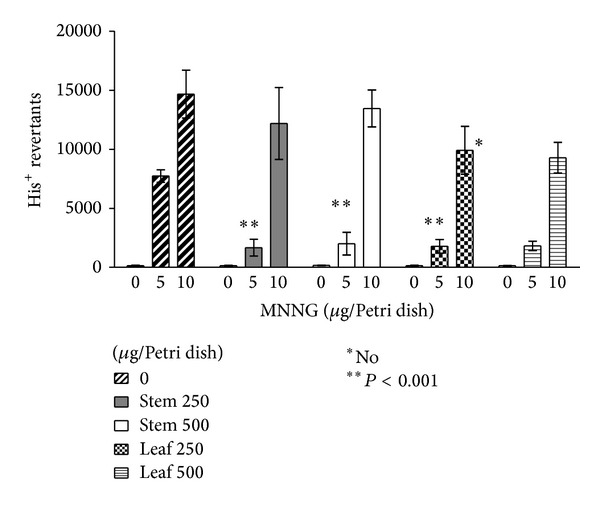
Antimutagenic effect of* A. californica* on methyl-N′-nitro-N-nitrosoguanidine- (MNNG-) induced mutations on* S. typhimurium* TA100.

**Figure 3:**
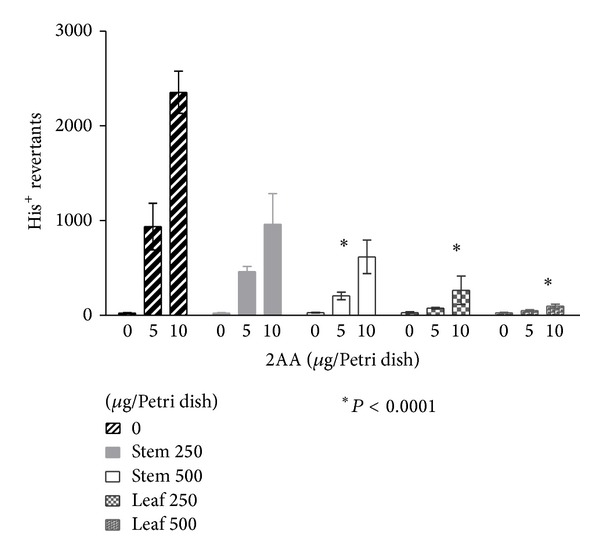
Antimutagenic effect of* A. californica* on 2AA-induced mutations and* S. typhimurium* TA98.

**Figure 4:**
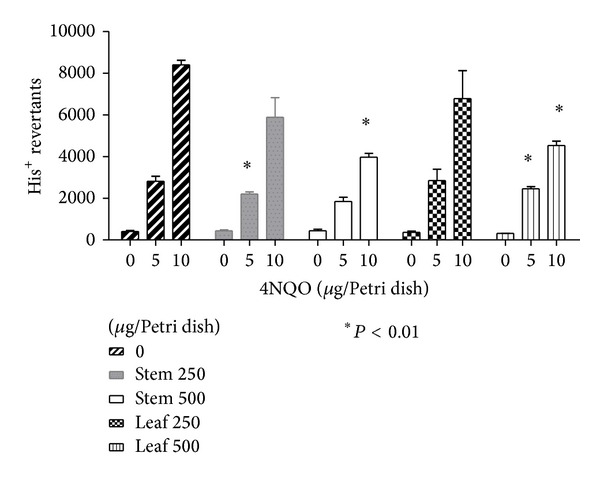
Antimutagenic effect of* Anemopsis californica* on 4NQO-induced mutations on* S. typhimurium* TA102.

LME and SME exhibited an antimutagenic effect. Mutations induced by MNNG on* S. typhimurium* TA100 were reduced only when 5 *μ*g of mutagen and 250 *μ*g of extract were tested which can be seen in Figure 2. In terms of the reduction of mutagenesis against higher mutagen concentrations, 10 *μ*g of MNNG was not statistically significant. The reduction of mutagenesis induced by the premutagen 2AA, activated with the S9 mix, was statistically significant (*P* < 0.001), even at the higher dose of premutagen 2AA (Figure 3). This reduction cannot be considered as a toxic effect because the wrought background on the Petri dishes was not damaged [27]. Reduction of ROS-induced mutations was also observed only when LME or SME were not exposed to higher concentrations of 4NQO (Figure 4).

## 4. Discussion

Phenolic compounds serve to improve the antioxidant properties of the plants; thus, in the majority of studies, they are correlated with antioxidant capacity [5]. In our study, the highest antioxidant capacity and total phenolic content were presented by SME. Little information concerning the bioactive compounds and antioxidant activities of the stems and leaves of* Anemopsis californica* grown in Mexico is available. On the other hand, this plant has been widely used and is empirically proven as a medicinal plant. However, there are not many studies on the safety of its use or the effects it may have on DNA. The results in this study show that* Anemopsis californica* did not induce any point mutations in the* S. typhimurium* Ames test. These results suggest that the LME and SME of* Anemopsis californica* may be safe for use in humans and should be considered for further medical development studies. In fact, the Ames test has been widely recommended as the first tool in the risk-exposure evaluation of therapeutic compounds or mixtures [10]. Mutagenicity evaluation for medical plants must be conducted, because not all naturally occurring compounds are innocuous.* Tacca integrifolia* Ker-Gawl, a plant used in Southern Asia, was found to be mutagenic on* S. typhimurium* strains TA98 and TA100. In Taiwan, betel quid, a natural chewing product composed of fresh green areca fruit, Piper betle, and slaked lime paste, is frequently used [32]. However, despite being a natural product, it demonstrated to be mutagenic on* S. typhimurium* TA100 because it contains N-nitroso guvacoline, an N-nitrosoguanidine. In Chile, it was found that red piper consumption increased gallbladder cancer. Scientific studies demonstrated that red piper was mutagenic on the Ames test and carcinogenic because it contained high doses of aflatoxins. In the present work, we observed that the extracts of* A. californica* isolated from Querétaro, Mexico, are not mutagenic.

Despite the fact that there are several mutagenic problems among naturally occurring plants used for their medicinal therapeutic properties or as food additives, the majority of these have been reported as antimutagenic rather than genotoxic.* Castela texana*, an antiamebic plant, was shown not to be mutagenic in the Ames test; in contrast, it is an antioxidant and reduces mutations induced by fluoroquinolones [2].* Rubia cordifolia* L. (Rubiaceae) is an important medicinal plant used in Ayurvedic medicine, especially for skin disorders.* Rubia cordifolia* contains alizarin, which is able to reduce the mutagenicity of 2AF as observed by the Ames test and damaged DNA induced by 4NQO on* Escherichia coli* [33].

In this study,* A. californica *induced a statistically significant increase (*P* < 0.001) of mutagenic properties in 2AA (Figure 3), a premutagenic compound that is a chemical carcinogen frequently found in air pollution. This significant effect is not considered toxic and no damage was observed on the background wrought in any of the plates. These results suggest that both extracts should be suitable for evaluation concerning CyP450 modulations effects. Another Mexican plant,* Heliopsis longipes*, and its main compound, affinin, were also able to reduce 2AA-induced mutagenicity [34]. The previous report from Kaur et al. [33] found antimutagenic properties in alizarin from* Rubia cordifolia* against the similar premutagen 2-aminofluorene (2AF). These results could be explained by finding that these medical plants might contain compounds capable of inhibiting the CyP450 required for activating these mutagens. In fact, Rodeiro et al. [35] reported that affinin reduced the expression of CyP450, CYP1A1/2, 2D6, and 3A4. In this study, we used aroclor-induced rat liver enzymes that contained CYP, which were necessary for the activation of polycyclic aromatic hydrocarbons.

The activity of this medicinal plant against the alkylating agent MNNG was observed only when the mutagen concentration was not very high (<5 *μ*g/Petri dish), as illustrated in Figure 2. These results are slightly different from the previously observed effects of the medicinal plant* Rhoeo discolor*, which significantly reduced basepair mutations when the* ogt* gene is present [4]. The compound responsible for the induction of* ogt* activity remains unknown.

In this work, we demonstrated that SME and LME of* A. californica* contain a high amount of polyphenols, which may possess several antimutagenic properties as had been reported throughout the literature. Schwarz et al. [36] evaluated that estradiol-dependent cancer might be regulated by polyphenols and the natural extract of St. John's Wort and several natural polyphenols. CYP1A1.1, responsible for estradiol metabolism, was most inhibited by the whole crude extract; the variant CYP1A1.2 (Ile462Val) was significantly more strongly inhibited by the constituents in its pure form. IC_50_ values for 2-hydroxylation were >2 times lower compared with the wild-type enzyme and the CYP1A1.4 variant (Thr461Asn). In addition, the inhibition exhibited remarkable region selectivity. The data suggest that the risk of estrogen-mediated diseases might be influenced not only by CYP1A1 genotype-dependent activation, but also by its inhibition by the natural polyphenols in our diet and drugs. In the present work, the whole extract of* A. californica* also shows a significant reduction in the mutagenicity of premutagens requiring CYP-450 metabolisms.

The results presented in this work show that LME and SME of* A. californica* possess antimutagenic properties against the ROS-generating compound 4NQO on the* S. typhimurium* TA102 strain. This strain is known to be sensitive to oxidative damage in DNA. It has been very useful in detecting ROS-generating DNA damage. The TA102 strain detects a variety of oxidative mutagens, including X-rays, bleomycin, hydrogen peroxide and other hydroperoxides, and 4-nitroquinoline-1-oxide [37]. Figure 3 shows that these extracts possess some antioxidant components; higher doses of both extracts induced a statistically significant reduction of 4NQO-induced mutagenesis (*P* < 0.01) especially when exposure to the mutagen is <10 *μ*g/Petri dish. This antimutagenic effect could probably be improved if the antioxidant component in these extracts could be purified. In fact, 4NQO is a potent mutagen that generates oxidative damage. It has also been employed as an inducer model of oral carcinogenesis with lymphatic metastasis in mice [38]. Miranda et al. [39] proposed that 4NQO-induced oxidative damage initiated the multistep process causing carcinogenesis in rat tongues. Polyphenols might probably be responsible for these effects. The literature on polyphenol antioxidant properties is abundant. In fact, Petriello et al. [40] recommend the employment of natural plants and plant-derived polyphenols as an alternative to reducing genotoxic risks to environmental pollution.

The antioxidant properties observed in the Ames test against 4NQO-induced mutations were also verified with the determination of antioxidant properties using the DPPH method, depending on the concentration of LME or SME used.

Antioxidant properties have been shown in other antimutagenic plants. It was previously found that* Castela texana* and* Rhoeo discolor *have antioxidant properties against the mutagenic effects of the ROS-generating quinolone antibiotics [2, 3]. It was demonstrated that 4NQO increased lipid peroxidation and decreased the levels of the nonenzymatic antioxidant compounds, as it reduced glutathione (GSH) and enzymatic antioxidants (superoxide dismutase (SOD), catalase (CAT), glutathione peroxidase (GPx), and glutathione-S-transferase (GST)) in both the liver and kidneys. Spirulina extracts were also antimutagenic against 4NQO-induced ROS damage [41].

## 5. Conclusions

*Anemopsis californica* did not induce any point mutations on the* S. typhimurium* Ames test, which renders it suitable for further studies because it possesses therapeutic uses. This medicinal plant has a slight antimutagenic effect against mutations induced by alkylating agents such as MNNG. It could provide protection against exposure to alkylating mutations. Additionally,* A. californica* as well as other medicinal plants possesses antioxidant properties. Its application could be useful for reducing genotoxic risk by exposure to ROS-generating exposure. Its most remarkable effect was found to be as an inhibitor of mutagenic induction of premutagens requiring CyP450, such as 2AA. This property demonstrates that the use of this medicinal plant could provide protection against polycyclic aromatic hydrocarbons which are well known as premutagens and precarcinogens.

## Figures and Tables

**Table 1:** Mutagenic activity of SME and LME.

Strain	SR	DMSO	DMSO + S9	SME (*μ*g/Petri dish)						LME (*μ*g/Petri dish)					
				1000		500		250		1000		500		250	
				Without S9	With S9	Without S9	With S9	Without S9	With S9	Without S9	With S9	Without S9	With S9	Without S9	With S9
TA98	22.0 ± 07.14	27 ± 01.41	29 ± 05.57	31 ± 04.16	37 ± 08.90	30 ± 08.84	30 ± 08.81	24 ± 06.50	24 ± 06.50	22 ± 03.60	44 ± 11.99	28 ± 02.80	28 ± 04.79	19 ± 02.00	30 ± 08.84
TA100	132.0 ± 16.66	155 ± 26.41	146 ± 17.67	148 ± 25.78	182 ± 16.30	—	—	—	—	177 ± 39.68	185 ± 26.24	—	—	—	—
TA102	305.0 ± 50.91	345 ± 67.22	322 ± 40.60	315 ± 60.78	356 ± 35.86	—	—	—	—	373 ± 55.50	396 ± 44.16	—	—	—	—

SME and LME: stem and leaf methanolic extracts, respectively. SR: spontaneous revertants. Positive controls: TA98: picrolonic acid (PA) (50 *μ*g/Petri dish) without S9, 475.0 ± 33.42, His^+^ revertants; (2AA) 2-aminoanthracene (10 *μ*g/Petri dish) with S9, 2354.83 ± 222.49; TA100: methyl-N′-nitro-N-nitrosoguanidine (MNNG), (10 *μ*g/Petri dish) without S9, 5,881.17 ± 861.49, (CF) cyclophosphamide (500 *μ*g/Petri dish) with S9, 530.0 ± 60.97; TA102: mitomycin C (2 ng/Petri dish) without, 3,401.67 ± 703.37, 2AA (10 *μ*g/Petri dish) 1,264.83 ± 77.38. S9 rat liver homogenate induced with aroclor 1254.
